# Increased DNA methylation, cellular senescence and premature epigenetic aging in guinea pigs and humans with tuberculosis

**DOI:** 10.18632/aging.203936

**Published:** 2022-03-07

**Authors:** Carly A. Bobak, Abhimanyu, Harini Natarajan, Tanmay Gandhi, Sandra L. Grimm, Tomoki Nishiguchi, Kent Koster, Santiago Carrero Longlax, Qiniso Dlamini, Jacquiline Kahari, Godwin Mtetwa, Jeffrey D. Cirillo, James O’Malley, Jane E. Hill, Cristian Coarfa, Andrew R. DiNardo

**Affiliations:** 1 Biomedical Data Science, Geisel School of Medicine, Dartmouth College, Hanover, NH 03755, USA; 2 The Global Tuberculosis Program, Baylor College of Medicine, Houston, TX 77030, USA; 3 William Shearer Center for Human Immunobiology, Texas Children's Hospital, Houston, TX 77030, USA; 4 Immigrant and Global Health, Department of Pediatrics, Baylor College of Medicine, Houston, TX 77030, USA; 5 Department of Microbiology and Immunology, Geisel School of Medicine at Dartmouth, Dartmouth College, Hanover, NH 03755, USA; 6 Dan L Duncan Comprehensive Cancer Center, Baylor College of Medicine, Houston, TX 77030, USA; 7 Molecular and Cellular Biology Department, Baylor College of Medicine, Houston, TX 77030, USA; 8 Department of Microbial Pathogenesis and Immunology, Texas A&M University Health, Bryan, TX 77807, USA; 9 Baylor-Swaziland Children’s Foundation, Mbabane, Swaziland; 10 The Dartmouth Institute, Dartmouth College, Hanover, NH 03755, USA; 11 Department of Chemical and Biological Engineering, University of British Columbia, Vancouver, BC, Canada

**Keywords:** tuberculosis, multi-cohort analysis, network analysis, DNA methylation, *Cavia porcellus*, senescence, DNA hypermethylation, epigenetic aging

## Abstract

Background: Tuberculosis (TB) is the archetypical chronic infection, with patients having months of symptoms before diagnosis. In the two years after successful therapy, survivors of TB have a three-fold increased risk of death.

Methods: Guinea pigs were infected with *Mycobacterium tuberculosis* (*Mtb*) for 45 days, followed by RRBS DNA methylation analysis. In humans, network analysis of differentially expressed genes across three TB cohorts were visualized at the pathway-level. Serum levels of inflammation were measured by ELISA. Horvath (DNA methylation) and RNA-seq biological clocks were used to investigate shifts in chronological age among humans with TB.

Results: Guinea pigs with TB demonstrated DNA hypermethylation and showed system-level similarity to humans with TB (*p*-value = 0.002). The transcriptome in TB in multiple cohorts was enriched for DNA methylation and cellular senescence. Senescence associated proteins CXCL9, CXCL10, and TNF were elevated in TB patients compared to healthy controls. Humans with TB demonstrate 12.7 years (95% CI: 7.5, 21.9) and 14.38 years (95% CI: 10.23–18.53) of cellular aging as measured by epigenetic and gene expression based cellular clocks, respectively.

Conclusions: In both guinea pigs and humans, TB perturbs epigenetic processes, promoting premature cellular aging and inflammation, a plausible means to explain the long-term detrimental health outcomes after TB.

## INTRODUCTION

Tuberculosis (TB), caused by *Mycobacterium tuberculosis* (*Mtb*), is responsible for about 1.4 million deaths worldwide each year [1]. TB is the archetypical chronic infection, with one-third of TB patients contending with the disease for a decade in the pre-antimicrobial era. Currently, TB patients have a median of three months of symptoms before diagnosis and retain a three-fold increased risk of mortality in the two years following successful therapy [2, 3]. In the US, premature death after successful treatment for TB was associated with seven years of life lost [4]. In a recent meta-analysis of over forty-thousand TB cases, mortality during TB treatment was 7%, and mortality in the two years after successful TB therapy was 16.9% [2].

Mouse models have demonstrated that in the setting of chronic infection, the epigenome rearranges to dampen host immunity and prevent host-inflicted immune pathology [5–7]. Specifically, the chronic antigenic stimulation results in histone deacetylation, histone methylation, and DNA hyper-methylation. Epigenetic modifications are a key process for the regulation of immune homeostasis as demonstrated in murine models of sepsis and chronic Lymphocytic choriomeningitis virus (LCMV), as well as human infections with HIV, TB schistosomiasis, and pneumonia [6, 8–11]. We recently demonstrated that TB patients develop DNA methylation changes similar to those seen in these animal models of chronic infection, with DNA hyper-methylation of genes involved in IL2-STAT5, TNF-NFκB, and IL12-IFNγ signaling pathways. Interestingly, these DNA hyper-methylation scars persist twelve months from initiation of antibiotics, six months from completion of successful TB therapy [8].

Our previous study in humans collected blood at the time of TB diagnosis, and therefore it is possible that these individuals already accumulated these detrimental epigenetic marks before TB. In this work using an established guinea pig (*Cavia porcellus*) model of TB, we clarify that TB induced DNA methylation changes that statistically overlapped with methylation changes observed in humans with TB. Thereafter, through untargeted network analysis of gene expression data, we demonstrated that TB induced gene expression changes that enriched for DNA methylation and other epigenetic processes in multiple cohorts, including people living with or without Human Immunodeficiency Virus (HIV), and in both adults and children. Network analysis of both gene expression and DNA methylation also confirmed that TB is associated with oxidative-stress induced senescence (OSIS) and the senescence-associated secretory pathway (SASP). By analysis of proteins shown to be elevated in SASP in an independent cohort from Eswatini, we confirmed that TB patients have increased SASP-related proteins [12] as well as increased Epigenetic and RNA-based age, with a mean increase from chronological age of 12.7 and 14.4 years respectively and a hazard ratio (HR) of 2.89 in all-cause mortality. In combination, the guinea pig and human data provide evidence that TB induces DNA hypermethylation, premature cellular aging, and inflammation.

## RESULTS

### TB induced DNA hypermethylation in guinea pigs, recapitulating global and specific features of human TB

**Figure 1:**
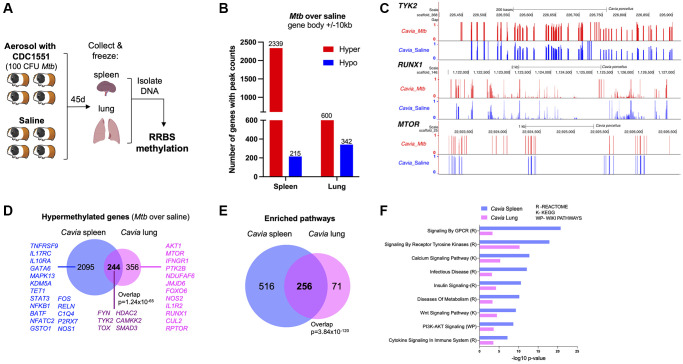
**Guinea pigs (*Cavia porcellus*) with TB exhibited DNA hypermethylation.** (**A**) Infection experimental design; guinea pigs were infected with 100 CFU of *Mtb* CDC1551. Forty-five days later, spleen and lungs were removed with DNA methylation evaluated by RRBS. (**B**) *Cavia* with TB have DNA hypermethylation in lung and spleen when compared to uninfected controls. The number of genes with hypermethylation (red) or hypomethylation (blue) are plotted for each tissue (within 10kb from DMRs). (**C**) Genome browser (UCSC) view of a few key hypermethylated genes in *Cavia* with TB (red bars) as compared to non-infected “Saline” (blue bars). The bar plots represent methylation values from a scale of ‘0’ unmethylated (black horizontal axis) to ‘1’ fully methylated. Overall mean values combining both spleen and lungs are plotted. The *Cavia* scaffold position after alignment is indicated on top for each gene. (**D**) Shared and unique hypermethylated genes between lung and spleen. (**E**) Overlap of enriched pathways between *Cavia* spleen and lung (based on KEGG, Reactome, and Wikipathways) using hypermethylated genes. (**F**) Selected common pathways relevant to TB disease with their –log10 *p*-value of enrichment.

Guinea pigs (*Cavia porcellus*) are a standardized animal model for TB, with many features that replicate human disease [13]; therefore, we evaluated the DNA methylation differences between TB diseased and control animals (*n* = 4). Methylation status of DNA isolated from spleen and lung tissue was evaluated 45 days post-infection (Figure 1A). Compared to uninfected controls, guinea pigs with TB had DNA hypermethylation of 2339 genes (80% of differentially methylated genes) in the spleen and 600 genes (60% of differentially methylated genes) in the lung (over 10% methylation ratio change and *p* < 0.05; Figure 1B; Supplementary Table 1). Genes critical to mycobacterial immunity, such as *NFKB1*, *TYK2*, *RPTOR*, *IL1R1* and *TOX,* were hypermethylated in either lung or spleen (Figure 1C, 1D). Spleen and lung shared 244 differential hypermethylated genes (DHG) (*p*-value of overlap = 1.24 × 10^−65^, Figure 1D) and 256 hypermethylated pathways (*p*-value of overlap = 3.84 × 10^−120^, Figure 1E). The common hypermethylated pathways in the lungs and spleen of guinea pigs included cytokine signaling, calcium signaling, metabolism, PI3K-AKT signaling, and tyrosine kinase pathways (Figure 1F).

**Figure 2:**
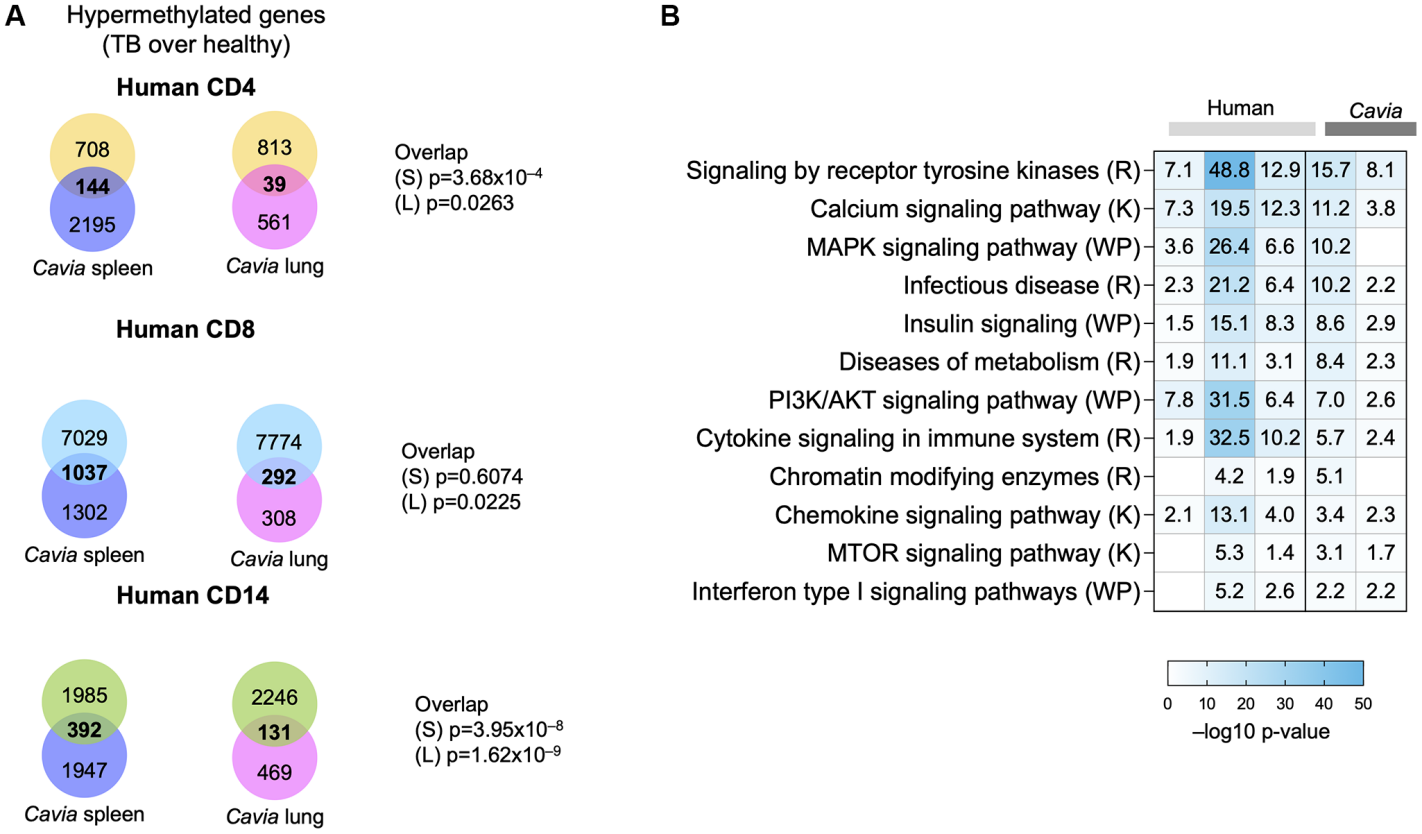
**Guinea pigs (*Cavia porcellus*) with TB exhibited systems-levels similarity with humans with TB.** (**A**) Venn diagrams depicting the overlap between genes with DNA hypermethylation in guinea pig spleen (blue circle), lungs (pink circle) and humans with TB (CD4, yellow circle; CD8, blue circle; CD14, green circle). The *p*-value of overlap are shown on the side for ‘S’: Spleen; ’L”: Lung. (**B**) Pathway enrichment analysis (MsigDB GSEA) demonstrating overlap in hypermethylated pathways in humans (light grey bars) and guinea pigs (dark grey bars) with TB. The box colors demonstrate –log 10 *p*-value of enrichment, with darker shades of blue indicating significance, with the −log10 *p*-values written in the square. Selected TB-relevant pathways are depicted.

We previously described DNA hypermethylation in humans with TB [8]. When comparing the DNA hypermethylation changes between humans and guinea pigs with TB, there was a statistically significant overlap with peripheral blood in human CD4, CD8 and CD14 cells (Figure 2A, Kolmogorov-Smirnov *p*-value = 0.002). Both humans and guinea pigs with TB demonstrated DNA hypermethylation of genes involved in pathways for the immune system, MAPK, tyrosine kinase, mTOR, calcium signaling, metabolism, and chromatin modifying enzymes (Figure 2B; Supplementary Table 2). Similarly, both humans and guinea pigs shared epigenetic changes in genes targeted by immune-related transcription factors (TF), including NFKBIA, TCF7, CIITA, MYC, NFAT and DNMT1/3A (Supplementary Figure 1; Supplementary Table 3). The TF were predicted to be regulating multiple genes across the groups (Supplementary Figure 1), establishing a systems-level overlap between human TB and guinea pigs. We previously demonstrated that TB-associated DNA hypermethylation in humans statistically overlapped with immune exhaustion, as induced by chronic infection with clone 13 LCMV in mice [8, 10, 11]. This was further validated by the DNA hypermethylation changes in guinea pigs enriching for targets of transcription factors, such as NFAT, MYC, TCF7, and NFKBIA (Supplementary Figure 1; Supplementary Table 3), which have been shown to lead to an immune inhibitory phenotype [14]. Combined gene and pathway analysis, using Metascape [15], demonstrated an interaction network enriched between humans and guinea pigs, including pathways for MAPK signaling, endocytosis, antigen presentation, calcium transport, cell adhesion, O-glycosylation of proteins, and chromatin modifying enzymes (Supplementary Figure 2).

### Enrichment of epigenetic regulators in human TB gene expression

To orthogonally validate the results, gene expression studies were also evaluated. Three datasets were evaluated based on their differing epidemiology. GSE42834 included adults with TB that excluded people living with HIV [16]; GSE37250 included adults with TB also living with HIV [17]; GSE39940 included children with TB [18]. The expression array datasets were collected from studies aiming to identify gene signatures that discriminated TB both from healthy controls as well as from other diseases, such as sarcoidosis, pneumonia, cancer, and other diseases that can mimic the clinical presentation of TB.

**Figure 3:**
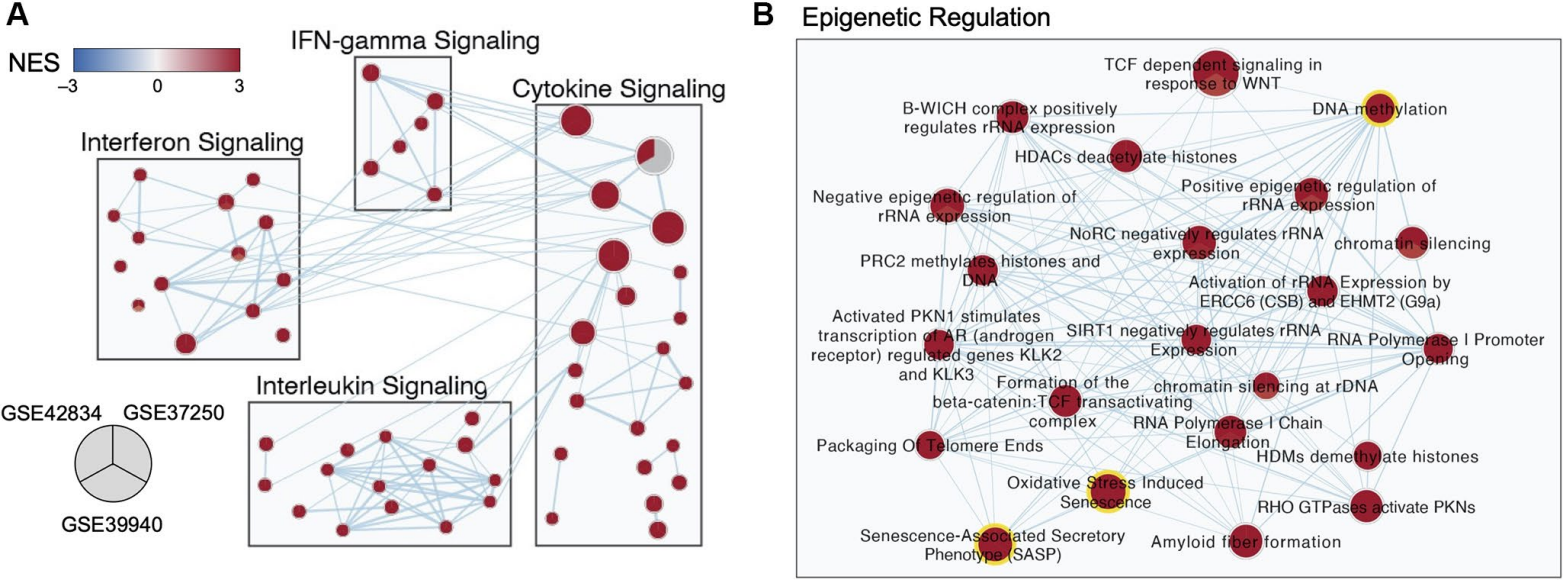
**Multi-cohort transcriptomic analyses corroborated a role for epigenetic regulation in TB.** (**A**) Selected subnetworks of enriched pathways associated with active TB diagnosis from three transcriptomic datasets. (**B**) Zoom-in of the Epigenetic Regulation Subcluster. Each node in the network represents an annotated gene set. Each node is a pie-chart corresponding to the normalized enrichment score (NES) for each dataset. Only nodes with a false discovery rate (FDR) *q*-value <0.01 in at least one dataset are depicted. Edges represent the overlap of genes between gene sets, where only overlaps >0.55 are visualized. The color of each pie on the map indicates the NES (blue for negative NES, and red for positive NES). The size of each node is proportional to the size of the gene sets.

Differentially expressed genes and pathways were visualized as a network of pathway-level results, with major pathway groups derived from an enrichment map (Figure 3). Subnetworks relevant to TB disease are shown in Figure 3A, the full network is available in [19].

An abundance of genes involved in modifying the epigenome were differentially expressed in the discovery datasets, including regulators of acetylation such as the histone deacetylases (*HDAC3, 1,5*), the NAD-dependent deacetylases Sirtuins (*SIRT1,2,4,5*), regulators of methylation such as DNA methyltransferases (*DNMT3A*), lysine demethylases (*KDM6B*), and chromatin modifiers such as the polycomb repressor complex components EZH2 and SUZ12 (Supplementary Figure 3). Previous studies have demonstrated that epigenetic changes can be triggered by oxidative stress and senescence-associated pathways [20–23] which were also enriched in all three TB gene expression datasets (Figure 3B).

**Figure 4:**
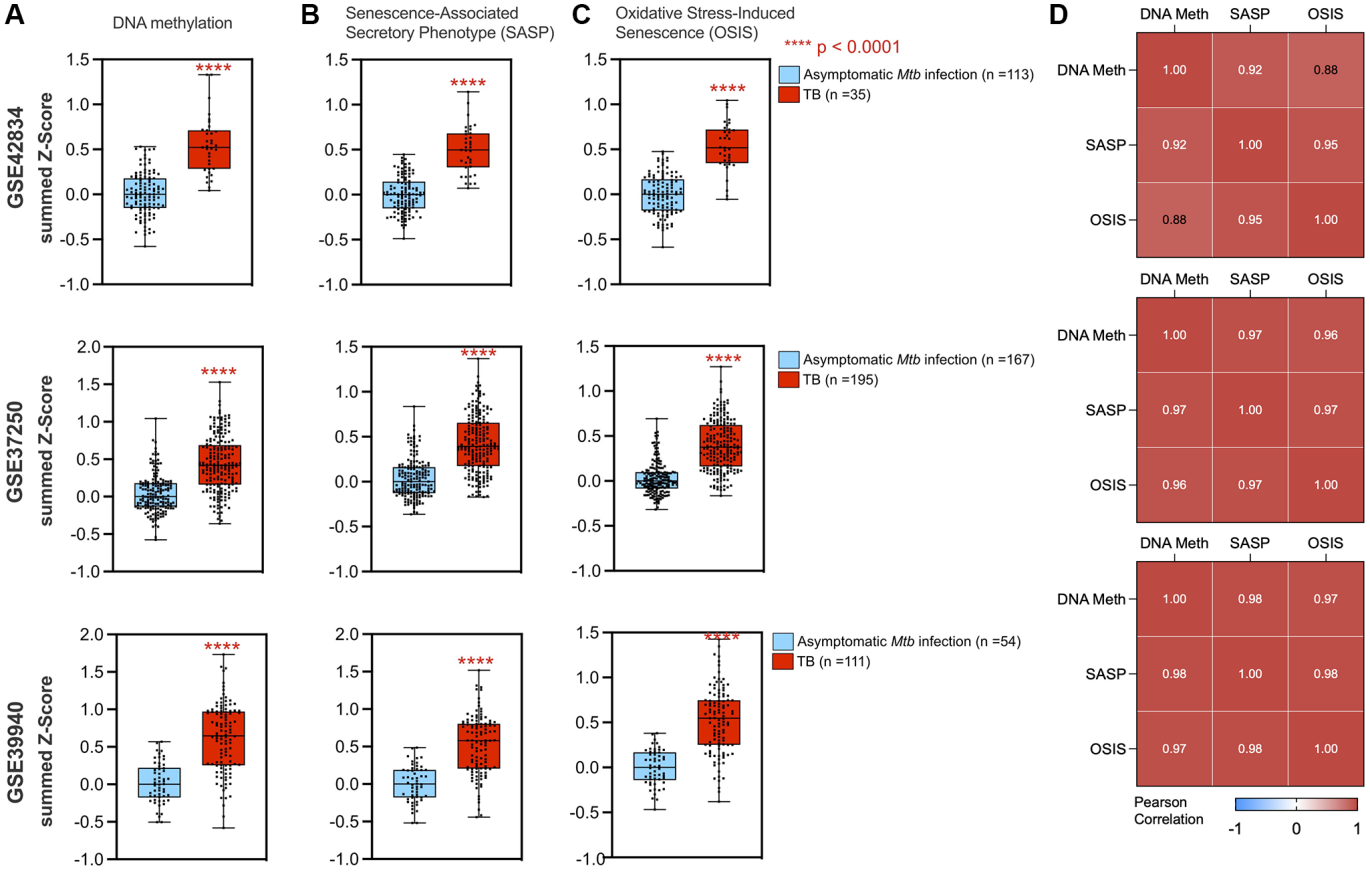
**DNA methylation and cellular senescence genes are increased and correlated in TB.** A summed z-score for gene expression from each patient was assessed for pathways including DNA methylation (**A**), SASP (**B**), and OSIS (**C**), with all three studies demonstrating increased summed z-scores in TB patients (red box plot) as compared to controls (blue box plot). *P*-values from a Wilcoxon rank sum test are indicated by asterisks. (**D**) DNA methylation correlated with senescence pathways using Pearson correlation for GSE42834, GSE37250, and GSE39940 respectively. Abbreviations: SASP: Senescence-associated secretory phenotype; OSIS: Oxidative stress-induced senescence.

Within the epigenetic regulation network, the DNA methylation node is a hub with 20 connections to other gene sets (degree = 20), including the oxidative stress-induced senescence and senescence-associated phenotype (Figure 3B). Across the three datasets, gene set enrichment analysis (GSEA) for DNA methylation genes had normalized enrichment scores (NES) of 2.10, 2.32, and 1.89 (adjusted *p*-value of 1.9 × 10^−5^, 3.9 × 10^−8^, and 6.0 × 10^−4^) for GSE42834, GSE37250, and GSE39940 respectively. From healthy controls, there was an increase in the DNA methylation summed z-score (Figure 4A). The most notable differences in signature Z-score occurred between samples from patients with TB and healthy controls or people with asymptomatic *Mtb* infection (*p* = 1.7 × 10^−11^, < 2.9 × 10^−26^, 3.5 × 10^−14^ in GSE42834, GSE37250, and GSE39940 respectively). We observed graded separation comparing TB disease to other diseases across the three distinct cohorts, substantiating that DNA methylation-associated gene changes due to TB disease is reflective of prior work in other chronic infections (Supplementary Figure 4) [5, 7]. The summed z-score for SASP and OSIS also was increased in TB patients compared to healthy controls or asymptomatic *Mtb* infected controls (*p* = 1.0 × 10^−14^, 7.3 × 10^−38^, 4.4 × 10^−14^ in the SASP pathway, *p* = 1.3 × 10^−14^, 6.8 × 10^−32^, 7.5 × 10^−16^ in the OSIS pathway for GSE42834, GSE37250, GSE39940 respectively; Figure 4B–4C). Correlation of DNA methylation, SASP, and OSIS pathway Z-scores was observed across all datasets (DNA methylation and OSIS r = 0.88, 0.96, and 0.97; DNA methylation and SASP r = 0.92, 0.97, 0.98; OSIS and SASP r = 0.95, 0.97, 0.98 for GSE42834, GSE32750, GSE39940 respectively; Figure 4D).

### TB induced premature epigenetic aging

**Figure 5:**
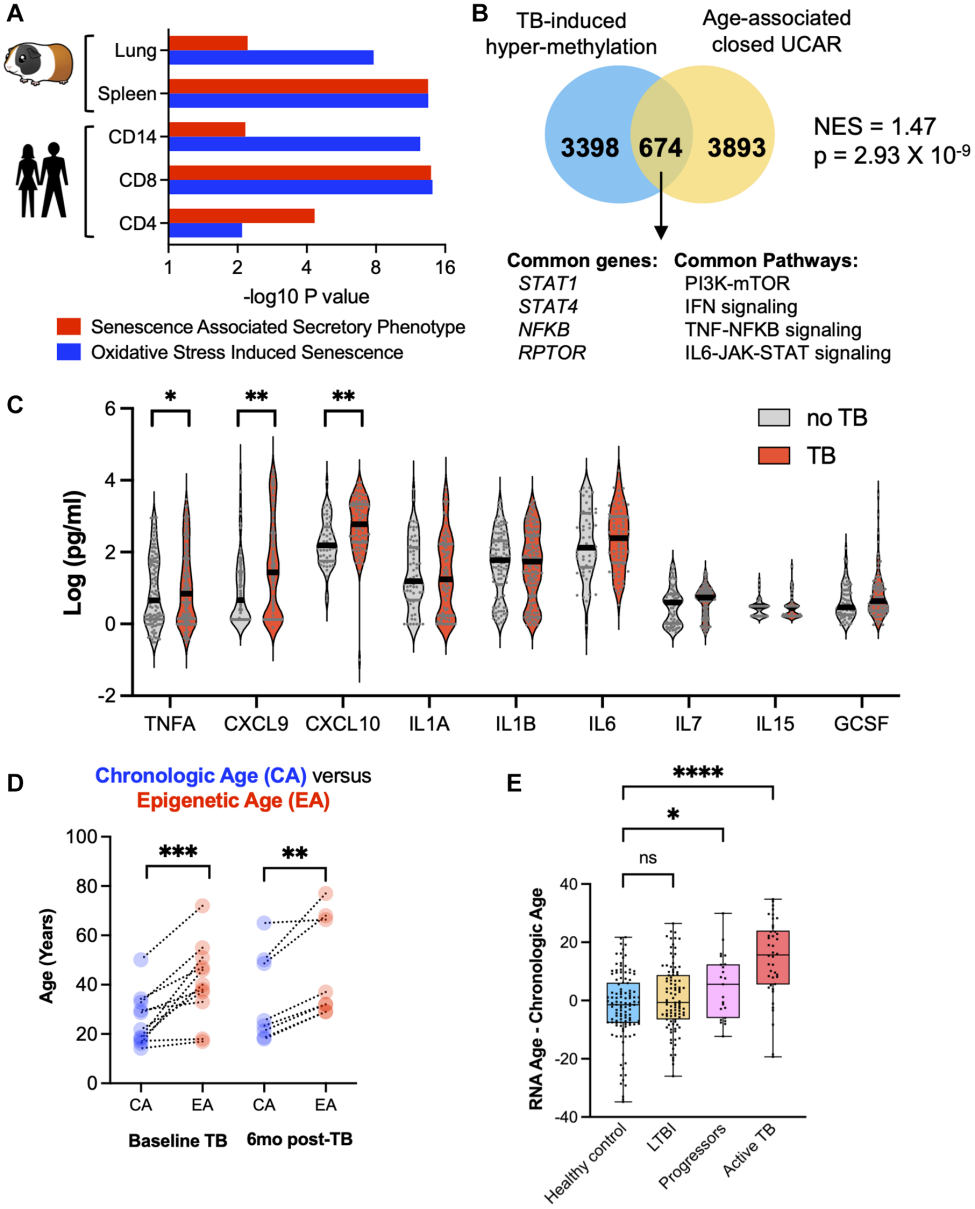
**TB induced cellular senescence and premature cellular aging.** (**A**) Humans and guinea pigs with TB demonstrated DNA hypermethylation gene changes that enriched for the SASP and OSIS pathways (Reactome overrepresentation *p*-values). (**B**) Hypermethylated genes in CD8+ T cells from patients with TB statistically overlapped with old age-associated closed chromatin conformation changes. (**C**) Multiplex ELISA of senescence associated proteins in patients with TB compared to healthy controls. (**D**) Epigenetic age (using the Horvath DNA methylation clock) is increased as compared to chronological age in TB patients at baseline and 6 months after the completion of successful anti-TB therapy. (**E**) Difference between chronological age and biological age using the RNA age calculator demonstrates an increase in TB patients compared to healthy controls (one-way ANOVA with Tukey’s multiple comparison).

In both humans and guinea pigs with TB, genes enriching for the SASP and OSIS pathways were hypermethylated compared to controls (Figure 5A). TB patients demonstrated a statistically significant enrichment of hypermethylated genes previously identified to have closed chromatin as part of the normal aging process [21] (NES = 1.47, *p* = 2.93 × 10^−9^; Figure 5B). At the protein level, in an independent cohort from Eswatini, TB patients demonstrated an increase in CXCL9, CXCL10, and TNFα, all components of the SASP (Figure 5C). Considering these findings, and the fact that aging is associated with increased DNA methylation, increased circulating senescence associated inflammation, and oxidative stress, we compared the biological age against the chronological age using two distinct biological clocks. The “Horvath clock” calculates biological age based on DNA methylation and has previously identified that HIV induced premature cellular aging [24, 25]. When the Horvath clock was applied to TB patients, there was an average increase in the DNA methylation age by 12.7 years above the chronological age (Figure 5D; Wilcoxon *p* < 0.0001, 95% CI (7.5–21.9 years)). This increased epigenetic age persisted at least 12 months from TB diagnosis (6 months after the completion of successful therapy; Figure 5D). To evaluate cellular aging using an orthogonal approach, a recently developed transcriptomic (RNA-sequencing based) clock ‘RNAAgeCalc’ was applied to an RNA-seq dataset (GSE107993; Figure 5E) [26]. Compared to healthy contacts and patients with asymptomatic *Mtb* infection (also known as latent *Mtb* infection; LTBI), the transcriptomic clock of TB patients was increased 14.38 years (95% CI: 10.22–18.53) over the chronological age (*p* < 0.0001, ANOVA with Tukey’s multiple comparison); Figure 5E). Moreover, individuals who were initially asymptomatic, but progressed to active TB disease within 6 months also demonstrated an increased transcriptomic clock of 4.83 years (95% CI: 0.10–9.56), which was significantly higher than healthy contacts and patients with asymptomatic *Mtb* infection (*p* = 0.0115, ANOVA with Tukey’s multiple comparison; Figure 5E). DNA methylation has been shown to be a strong predictor of all-cause mortality [27], including an increase in Horvath epigenetic age. Considering the Horvath age increase of 12.7 years, TB cases computed as per Zhang et al. [27] had an increased hazard ratio (HR) of 2.89 (95% CI: 2.40–3.12) in all-cause mortality.

## DISCUSSION

At the completion of apparently successful therapy, TB patients have a nearly 3-fold increased risk of death due to unknown reasons [2, 4]. Our previous work demonstrated that TB patients exhibited DNA hypermethylation associated with decreased immune responsiveness and that the DNA hypermethylation marks did not normalize six months after the completion of successful TB therapy [8]. Using a guinea pig model, this study corroborates that TB induces DNA hypermethylation marks and that the epigenetic perturbations in guinea pigs recapitulated human disease. Further, at transcriptomic and epigenomic levels, both guinea pigs and humans with TB enriched for senescence and oxidative stress induced senescence pathways, with humans afflicted with TB demonstrating more than a decade of premature cellular aging by both epigenetic and transcriptomic clocks. Increases in DNA hypermethylation and cellular senescence are plausible mechanisms for the long-term increased risk of death in TB patients and, as such, they need to be prospectively evaluated.

In order to temper exuberant, pathologic immunity in the setting of chronic or severe infection, epigenetic mechanisms dampen both adaptive and innate immunity to prevent collateral host tissue destruction [5, 6, 28]. Unfortunately, these epigenetic scars are long-lived, thereby increasing the risk for secondary infections [7]. Both severe and chronic infections perturb host epigenetic regulation, thereby suppressing host immunity [29–31]. HIV also perturbs host epigenetic regulation, resulting in long-lasting DNA methylation changes in IL-2, PD1, and the regulation of IFN-γ [32, 33].

Previously, it was unclear if individuals with DNA hypermethylation were at increased risk of progressing to TB, or if TB itself was inducing DNA hypermethylation. While the options are not mutually exclusive, the guinea pig studies lucidly demonstrated that TB induces DNA hypermethylation. This is also supported by the orthogonal gene expression analysis, demonstrating an increase in genes related to DNA methylation among TB patients. This does not exclude the possibility that individuals with increased DNA methylation are at increased risk of progression to TB. In fact, the gene expression analysis suggests that multiple diseases induce DNA methylation perturbations. Both schistosomiasis and HIV induced DNA hypermethylation and both are associated with increased risk of TB disease progression [33, 34]. Further, compared to asymptomatic controls that stayed healthy, individuals that were originally asymptomatic, but then progressed to overt TB disease, had an increase in premature aging as calculated by the RNA clock. Plausibly, comorbidities like air pollution, smoking, schistosomiasis, and HIV result in DNA hypermethylation, suppressing the host immune response, and therefore increasing the risk of TB disease progression. Future experiments will need to evaluate if preceding unrelated infection would further predispose risk for TB progression due to epigenetic perturbations.

After successful therapy for pneumonia, sepsis, or TB, there remains an increased risk of mortality [4, 35–37]. A recent retrospective study examining 2522 TB patients in the United States calculated an average of 7.0 (95% CI: 5.5–8.4) years of potential life lost despite apparently successful therapy [4]. Similarly, we calculated a nearly 3-fold risk (HR = 2.89) in all-cause mortality in TB patients using predictors described by Zhang et al. [27]. The observations that cellular senescence is enriched in both gene expression and DNA methylation datasets of patients with TB, and the correlation between methylation and senescence need to be mechanistically explored to identify if the >10 years of premature cellular aging is reversible. Additional evidence supporting TB induced “inflammaging” includes elevations in plasma levels of SASP markers such as TNFα, CXCL9, and CXCL10, providing additional evidence that long-term health and mortality is negatively impacted by the development of TB disease. This phenomenon has previously been observed in people living with HIV in a study conducted by Horvath et al. [24]. The increase in pathways of DNA methylation, oxidative stress induced cellular senescence, and senescence associated secretory phenotype summed z-scores, and correlation between these pathways, suggests there are common epigenetic perturbations that occur with chronic inflammation, offering optimism that there may be common pathways to remediate these changes. Further understanding of the role of accelerated aging in TB patients may be also elucidated by decreased telomere length and increased mitochondrial DNA copy number in TB patients [38].

An interesting observation was the lower number of changes observed in the lungs as compared to the spleen in the guinea pigs. Although we see a significant overlap between post-infection methylation changes between the two organs, our analysis reveals a tissue-specific response to pathogens, which may partially be explained by the distinct cell type composition of the two tissues.

While the results of the DNA methylation studies were limited by the small sample sizes in the guinea pigs, this would still allow for the observation of large effect sizes. Further, the findings were corroborated using orthogonal transcriptomic and protein analyses. We also see statistically significant overlap with the previous DNA hypermethylation findings in human studies suggesting that even the small size of the guinea pig model was able to capture major meaningful perturbations in the epigenome. Future examinations will need to validate these observations in larger numbers in both guinea pigs and humans, with particular focus on the commonly perturbed pathways like PI3K/AKT or MTOR signaling. Experiments suggest that immune inhibitory epigenetic marks were triggered by specific events (NFAT homodimerization, intracellular metabolic shifts) that resulted in histone deacetylation, DNA hypermethylation, and chromatin closing [39, 40]. A longitudinal approach should be considered to examine if increases in DNA methylation or premature aging are associated with increased risk of TB disease progression and, vice versa, if DNA methylation and premature aging improve after successful therapy.

## CONCLUSIONS

This work, using multiple cohorts, multiple tissue types, and both transcriptomic and DNA methylation sequencing, provides evidence that TB induced perturbations in epigenetic regulation, specifically in DNA methylation that correlated with oxidative stress induced senescence and was associated with premature cellular aging, measured by both epigenomic and transcriptomic based clocks. These processes were conserved across both guinea pigs and humans, indicating that a guinea pig (*Cavia porcellus*) model may be appropriate for further mechanistic research. The TB induced premature aging is a plausible mechanism for increased risk of death that occurs after successful therapy for TB and needs to be prospectively evaluated.

## MATERIALS AND METHODS

### Animal aerosol challenge, determination of bacterial load, and DNA extraction

All guinea pig experiments were reviewed and approved by the Institutional Care and Use Committee at Texas A&M University in compliance with the National Institute of Health guidelines, as described in the Guide for the Care and Use of Laboratory Animals. Eight pathogen-free female Harley guinea pigs (250–300 g) were obtained from Charles River Laboratories. The animals were maintained with commercial diet and water *ad libitum.* The guinea pigs were divided into two groups of four, one group underwent aerosol infection with *Mtb* strain CDC1551, grown as described previously [41, 42] and washed with saline and suspended at an OD_600_ of 0.1 prior to nebulization using a Madison chamber, as described previously [41, 43]. The other group was used as a control without any infection procedure. Initial colony forming units (CFU) in the lungs were ~100 CFU. The average number of CFU present in the entire lungs at 45 days was 4.2 ± 0.7 × 10^6^ CFU, the CFU plateaued just after 45 days post-infection, which was hence chosen as the time point at which humane euthanasia was performed with pentobarbital (FatalPlus). At necropsy, organs were collected and frozen at −80°C until use. DNA was isolated from organs using the DNeasy blood and tissue kit (Qiagen Inc.), followed by nucleic acid quantity determination with a Qubit fluorometer using the dsDNA BR assay kit (Thermo Fischer Scientific, Waltham, MS, USA).

### Quantification of DNA methylation in guinea pigs and humans

DNA methylation from the spleen and lung of the control and TB guinea pigs was measured using reduced representation bisulfite sequencing (RRBS) as previously described [44, 45] to map the methylated cytosines in the DNA obtained from spleen and lung from infected and uninfected *Cavia porcellus*, as described in the previous section. Library preparation was performed using Ovation RRBS Methyl-Seq System kit (NuGEN Technologies, Inc, Redwood City, California). In brief, 100 ng of genomic DNA was digested with *Msp*I, and Illumina-compatible cytosine-methylated adaptors were ligated to the enzyme-digested DNA. Size-selected fragments were bisulfite-converted, and library preparation was done by PCR amplification, and subsequently sequenced in a HiSeq3000 instrument at the MDACC Epigenomics Profiling Core Facility. RRBS sequencing reads were aligned to *Cavia* reference genome and DNA methylation ratios at CpGs were called using Bismark v0.7.11 [46]. Next, differentially methylated CpG sites (DMCs) and differentially methylated regions (DMRs) between infected and uninfected *Cavia* were identified using BS-seq [47]. DMR-associated genes were determined using BEDTOOLS [48] and a window of 10,000 base pairs around coding gene bodies.

Peripheral blood mononuclear cells (PBMCs) from human subject samples, previously published, underwent DNA isolation using the Qiagen DNeasy kit, followed by bisulfite conversion [8]. DNA methylation was determined by the Illumina MethylEPIC array and was deposited in the NCBI Gene expression omnibus (GSE145714). The gene list was also published as a supplementary table, from which the information was obtained for the overlap analyses [8].

### Functional enrichment analysis for guinea pig and human methylation

To evaluate gene or pathway overlaps Venn diagrams were obtained using the online portal (http://bioinformatics.psb.ugent.be/webtools/Venn/). Gene set enrichment analysis was carried out using the hypergeometric distribution as implemented by the MsigDB online tool (http://www.gsea-msigdb.org/) against the following gene collections including hallmark, KEGG, Reactome and WikiPathways to compute overlaps with an FDR cut-off <0.05 for the significant genes from the guinea pig and human methylation datasets [49]. Overall system level similarly was compared using a two-sided Kolmogorov-Smirnov test, where overlap *p*-values were compared to the null hypothesis that they follow a uniform distribution. In addition, targets of transcription factor (TFT) overlaps were also computed for all lists using the same portal. Meta-pathway analysis was used to find gene and simultaneous pathway level overlap between the human and the guinea pig datasets through Metascape online pathway analysis portal (https://metascape.org/gp/index.html#/main/step1) [15]. Over-representation analysis for OSIS and SASP was carried out using the analysis option of the Reactome database, where the hypermethylated genes from humans and guinea pigs were used as input. The protein-protein interaction (PPI) network was generated using MCODE algorithm to identify enriched clusters, represented as a merged network visualized using Cytoscape [50, 51]. The network was then annotated using gene ontology (GO) enrichment analysis as described by Zhou et al. [15] The bar graphs were plotted using GraphPad Prism (version 9.1.0) for macOS [52].

### Quantification of the serum cytokines in a TB cohort

The plasma samples were obtained from a previously described cohort of adults with TB symptoms and microbiologically confirmed pulmonary TB (by culture and/or GeneXpert) from Eswatini. They were compared to with their asymptomatic, healthy household contacts who remained asymptomatic for 12 months after initial exposure [8]. The serum was evaluated by using nine soluble markers of senescence associated secretory phenotype as defined previously [53, 54], including IL15 (TB = 40, HC = 39), TNFA (TB = 152, HC = 111), IL1B (TB = 152, HC = 111), CXCL10 ( TB = 70, HC = 49), IL6 (TB = 70, HC = 49), CXCL9 (TB = 152, HC = 111), IL7 (TB = 112, HC = 72), GMCSF (TB = 82, HC = 62), IL1A (TB = 82, HC = 62) using a customized bead-based multiplex assay (LEGENDplex kits, BioLegend), according to manufacturer’s instruction. Briefly, the human plasma was diluted 1:1 and incubated with APC-conjugated capture beads, specific to each analyte being measured, supplied with the kit. Known standards for each analyte were also incubated in duplicate. Biotinylated detection antibodies were added which form capture bead-analyte-detection antibody sandwiches. Streptavidin-phycoerythrin (SA-PE) was subsequently added, providing fluorescent signal with intensities in proportion to the amount of bound analyte. For each bead population, the PE signal fluorescence intensity was quantified using a flow cytometer. The concentration of a particular analyte was determined based the known standard curve using the LEGENDplex data analysis software.

### Gene expression cohort selection

Gene expression array datasets were downloaded from the Gene Expression Omnibus (GEO) hosted by the National Center for Biotechnology Information (NCBI). Search terms to identify datasets included ‘Tuberculosis’ and ‘TB’ and were limited to whole blood samples. We selected three datasets based on the heterogeneity of the epidemiology. The first cohort included only adults and excluded HIV positive patients [16]. The second cohort included adults, both people with and those without HIV co-infection [17]. Finally, the last cohort included children, with and without HIV co-infection [18]. All three datasets were analyzed using the Illumina HumanHT12v4 bead chip. A total of 1152 samples were used in analysis. These studies were designed to identify a novel TB diagnostic test, and therefore they included diseases that present similar to TB, including other pneumonias, sarcoidosis, cancer, and other infections.

### Analysis of expression data

Analyses were conducted in R version 4.0.3. Normalized data were downloaded using the ‘GEOquery’ package, and checked to ensure that values were mean-centered and log_2_ transformed [55]. Differential expression (DE) analysis of genes was conducted using the ‘limma’ package in R to calculate the empirical Bayes (moderated) pooled variance for each gene where active TB was contrasted to all the other disease classes as a one-vs.-rest problem. DE analysis identified genes that were significantly associated (either positively or negatively) with the outcome of interest, in this case active TB disease.

The log_2_ fold change (FC) and *p*-values from the DE analysis were used to calculate scores and rank genes for Gene Set Enrichment Analysis (GSEA) using


$$Rank=sign(\log_{2}(FC)\times (-1)\log_{10}(p)\;\;(1)$$

as suggested in [56]. These ranks were input to GSEA Desktop v3.0 to identify which gene sets were enriched or depleted in each of the three data sets [57]. The Gene Ontology (GO) process collection of gene sets was downloaded from the Molecular Signature Database (MSigDB) [49]. GSEA compared gene sets and genes which were close to the top or the bottom of the ranked list to determine whether the gene set was over-represented. Each gene set was given a Normalized Enrichment Score (NES) [57]. The output after running GSEA was used to create an EnrichmentMap in Cytoscape v.3.6.0 [50, 58]. Subnetworks were identified using AutoAnnotate [59] and those related to epigenetic regulation are presented.

We evaluated the DNA methylation, oxidative stress induced cellular senescence, and senescence associated secretory phenotype genes using a pathway z-score, calculated as the as the difference of the mean of upregulated and the mean of downregulated genes contained in each pathway and measured in each dataset, as suggested in [60]. We centered pathway scores on the median value observed in the non-asymptomatic control group in each dataset. A Wilcoxon rank sum test was used to test if these pathway scores were significantly different between TB and healthy controls or other diseases [61]. We calculated the correlation between the observed z-scores of each of the epigenetic regulation pathways of interest within each dataset and used ‘pheatmap’ [62] to evaluate if correlations were being driven by overlapping genes contained within the pathways.

### Horvath methylation age prediction

The CpGs required for estimating the Horvath DNA methylation age [63] were derived from the published dataset and used in an R pipeline containing a modified version of Steve Horvath’s code (https://dnamage.genetics.ucla.edu) to perform normalization and estimate the biological age in human TB published data [8]. The statistical tests were run using GraphPad Prism (version 9.1.0) for macOS. All-cause mortality was derived based on Horvath age estimates as described [27]; briefly they estimated that for every 5-year increase in Horvath age the Hazard increases by 1.14. Increased risk was estimated by the formula: Increased Risk = ((Horvath age estimate)/5) × 1.14. The CI were multiplied by the same factor as the increased risk.

### RNA age prediction

We sought publicly available RNA Sequencing data on the GEO. To be eligible for analysis, the data needed to contain whole blood samples from participants with active TB samples collected from time of diagnosis, prior to treatment, as well as patient level chronological age. We selected the largest such dataset for the RNA Age prediction, GSE107993 [26]. We removed outliners, patients missing chronological age, and only used baseline samples across all disease categories. RNA age was calculated using the ‘RNAAgeCalc’ package in R [64]. We calculated RNA age using the raw sequencing counts, specified that our tissue was blood, our signature type was ‘all’, and used the Peter’s age signature. Considering the increase in biological age above chronological age as measured by DNA methylation, we calculated the difference between RNA and chronological age and used a one-way ANOVA with Tukey’s multiple comparison to evaluate if the age difference was significantly increased with TB disease compared to household contacts, LTBI, and future LTBI progressors.

## Supplementary Materials

Supplementary Figures

Supplementary Tables 1-3
