# Supplementary material for: Increased DNA methylation, cellular senescence and premature epigenetic aging in guinea pigs and humans with tuberculosis
Source: Aging (Albany NY). 2022 Mar 7;14(5):2174–93. doi: 10.18632/aging.203936 (PMC8954968; doi:10.18632/aging.203936)
Supplement: Supplementary Figures [file aging-14-203936-s001.pdf]

SUPPLEMENTARY FIGURES

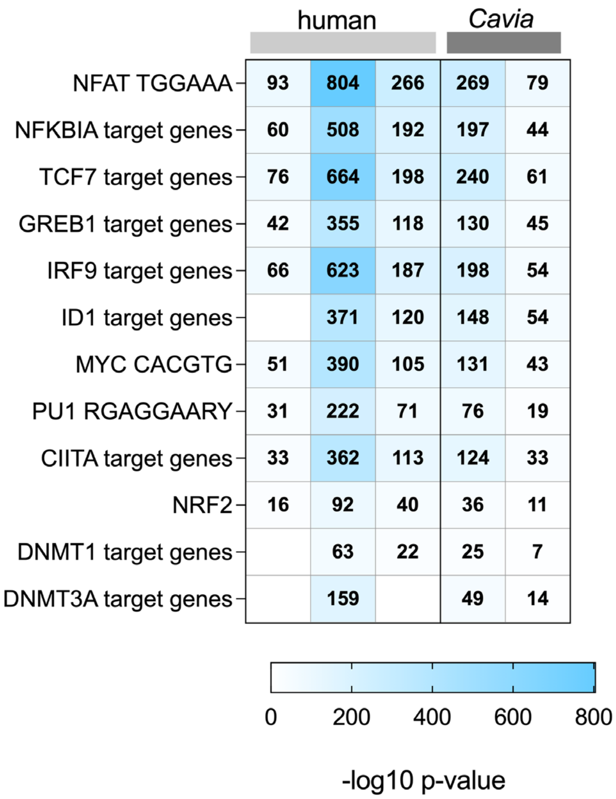

Supplementary Figure 1. TF overlap between guinea pigs and humans. MsigDB Gene set enrichment analysis shows considerable overlap in TF targets between humans (light grey bars) and guinea pigs (dark grey bars) with TB. The box colors show –log 10 *p*-value of enrichment, with darker shades of blue indicating significance. The numbers in the squares indicate the predicted gene count. Only a subset of TB-relevant TF targets are shown, for the full list refer to Supplementary Table 3.

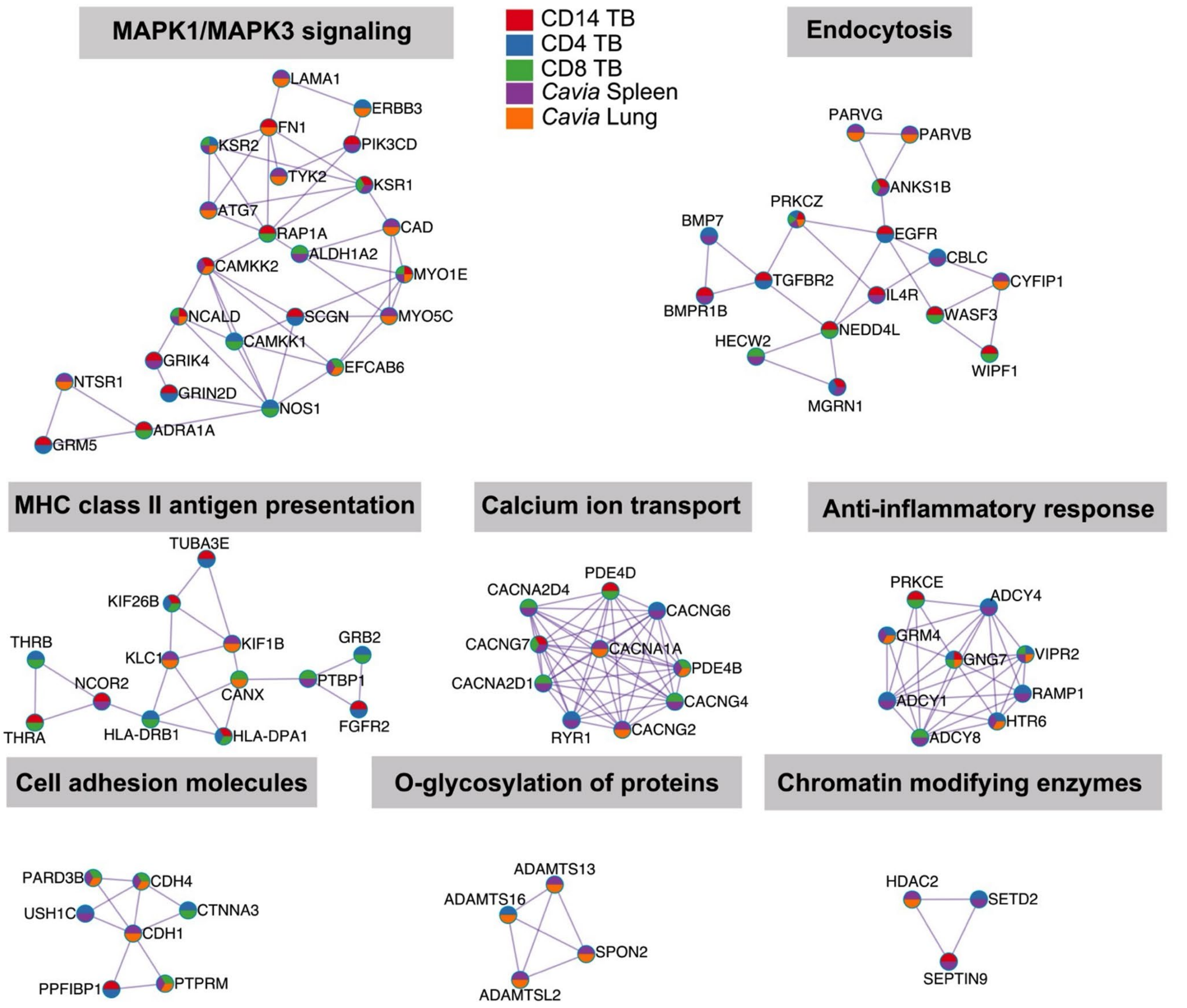

Supplementary Figure 2. Shared genes and pathway between guinea pigs and humans. Enrichment network generated in Metascape [15] from five gene lists, three from human data [8] and two from *Cavia* generated in this study. Top 1000 differentially hypermethylated genes from all merged gene list was used for analysis. The nodes represent pie charts, where the color represents the identity in the input gene list. Cluster labels are derived from functional enriched gene term for that cluster. If the same gene is shared between multiple lists, the color appears in the pie.

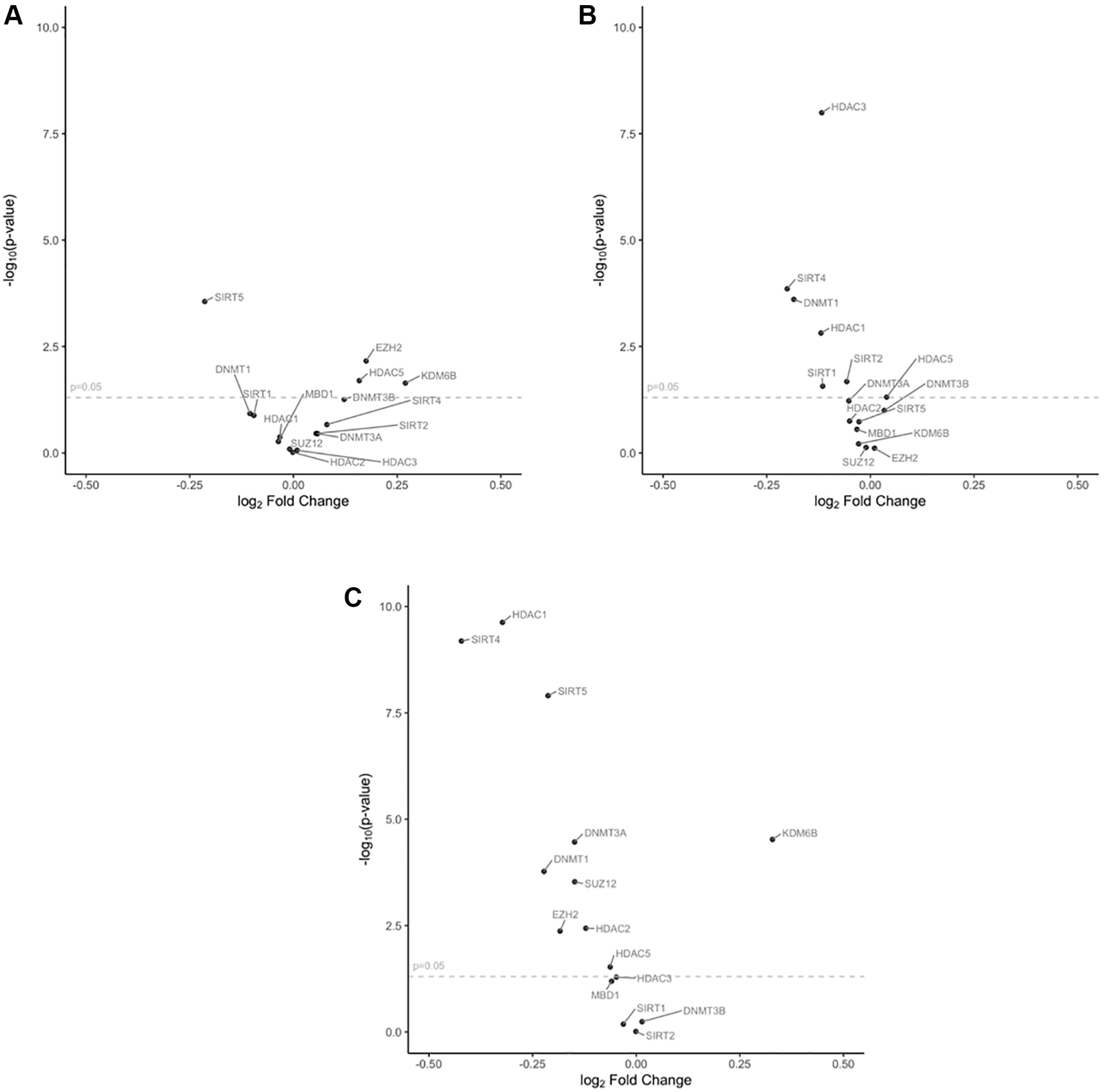

Supplementary Figure 3. Volcano plots showing the differential gene expression of canonical genes associated with epigenetic regulation in (**A**) GSE42834, (**B**) GSE37250, and (**C**) GSE39940. Differential expression (DE) analysis of genes was conducted using the 'limma' package in R to calculate the log_2FC and *p*-value of each gene where active TB was contrasted to all the other disease classes as a one-vs.-rest problem.

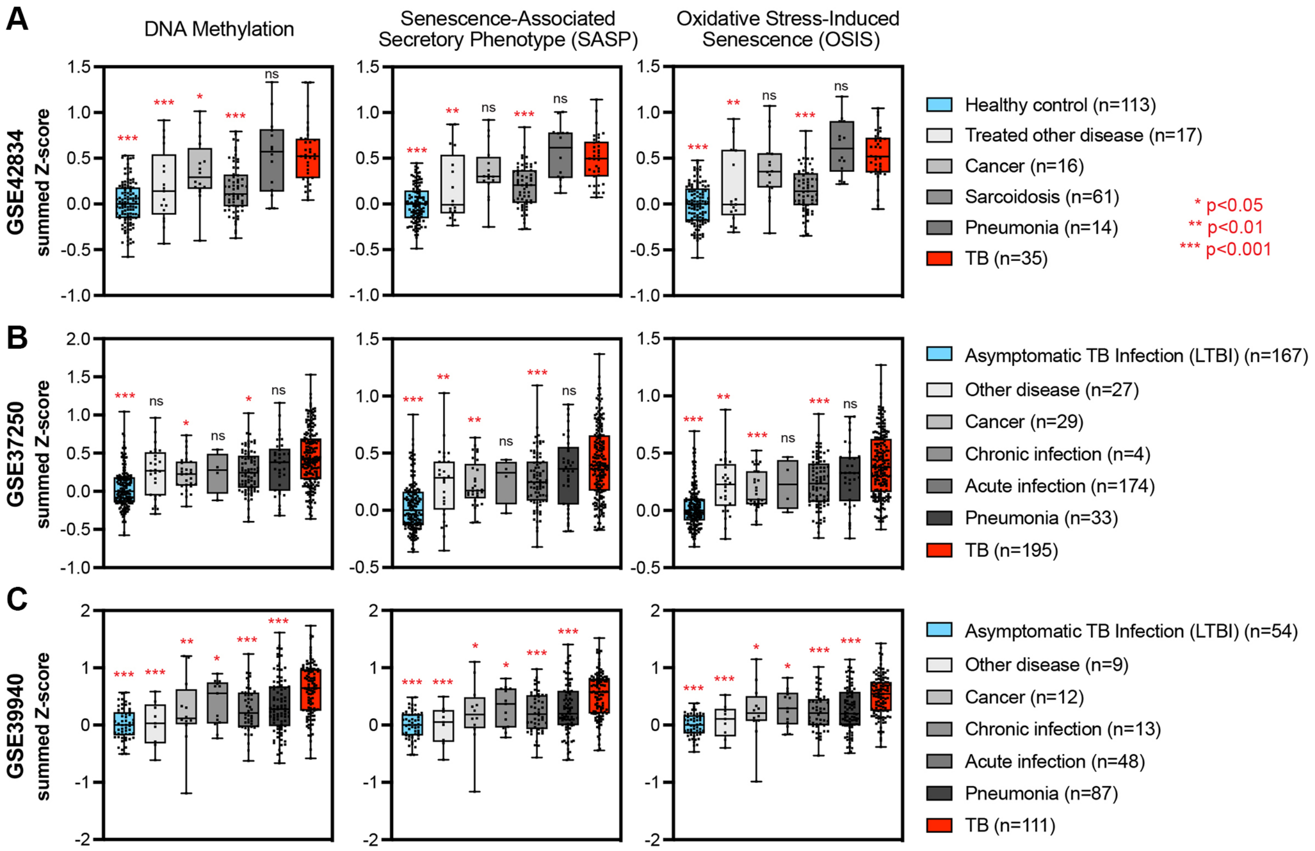

Supplementary Figure 4. DNA methylation and cellular senescence genes are increased TB and other chronic infections. (**A**–**C**) A summed z-score for gene expression from each patient was assessed for pathways including DNA methylation, SASP, and OSIS pathways, with all three studies demonstrating increased summed z-scores in TB patients (red box plot) as compared to other groups. *P*-values from a Wilcoxon rank sum test are indicated by asterisks. Abbreviations: SASP: Senescence-associated secretory phenotype; OSIS: Oxidative stress-induced senescence.
